# Lymph node CXCR5^+^ NK cells associate with control of chronic SHIV infection

**DOI:** 10.1172/jci.insight.155601

**Published:** 2022-04-22

**Authors:** Sheikh Abdul Rahman, James M. Billingsley, Ashish Arunkumar Sharma, Tiffany M. Styles, Sakthivel Govindaraj, Uma Shanmugasundaram, Hemalatha Babu, Susan Pereira Riberio, Syed A. Ali, Gregory K. Tharp, Chris Ibegbu, Stephen N. Waggoner, R. Paul Johnson, Rafick-Pierre Sekaly, Francois Villinger, Steve E. Bosinger, Rama Rao Amara, Vijayakumar Velu

**Affiliations:** 1 Division of Microbiology and Immunology, Emory Vaccine Center, Yerkes National Primate Research Center, Emory University, Atlanta, Georgia, USA.; 2 Department of Microbiology and Immunology and; 3 Department of Pathology and Laboratory Medicine, Emory University School of Medicine, Atlanta, Georgia, USA.; 4 New Iberia Research Center, University of Louisiana at Lafayette, New Iberia, Louisiana, USA.; 5 Department of Pediatrics, University of Cincinnati College of Medicine, Cincinnati, Ohio, USA.; 6 Infectious Disease Division, Department of Medicine, Emory University School of Medicine, Atlanta, Georgia, USA.

**Keywords:** AIDS/HIV, Immunology, Innate immunity, NK cells

## Abstract

The persistence of virally infected cells as reservoirs despite effective antiretroviral therapy is a major barrier to an HIV/SIV cure. These reservoirs are predominately contained within cells present in the B cell follicles (BCFs) of secondary lymphoid tissues, a site that is characteristically difficult for most cytolytic antiviral effector cells to penetrate. Here, we identified a population of NK cells in macaque lymph nodes that expressed BCF-homing receptor CXCR5 and accumulated within BCFs during chronic SHIV infection. These CXCR5^+^ follicular NK cells exhibited an activated phenotype coupled with heightened effector functions and a unique transcriptome characterized by elevated expression of cytolytic mediators (e.g., perforin and granzymes, LAMP-1). CXCR5^+^ NK cells exhibited high expression of FcγRIIa and FcγRIIIa, suggesting a potential for elevated antibody-dependent effector functionality. Consistently, accumulation of CXCR5^+^ NK cells showed a strong inverse association with plasma viral load and the frequency of germinal center follicular Th cells that comprise a significant fraction of the viral reservoir. Moreover, CXCR5^+^ NK cells showed increased expression of transcripts associated with IL-12 and IL-15 signaling compared with the CXCR5^–^ subset. Indeed, in vitro treatment with IL-12 and IL-15 enhanced the proliferation of CXCR5^+^ granzyme B^+^ NK cells. Our findings suggest that follicular homing NK cells might be important in immune control of chronic SHIV infection, and this may have important implications for HIV cure strategies.

## Graphical abstract

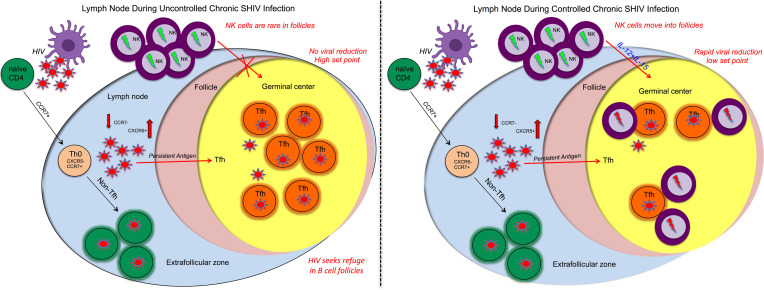

## Introduction

The persistence of HIV/SIV reservoirs under combined antiretroviral therapy (ART) remains an obstacle to achieving sustained viral control upon ART discontinuation (1, 2). HIV/SIV has been shown to readily spread to the B cell follicles (BCFs), where follicular Th cells (Tfh cells) are preferentially infected and serve as viral reservoirs under ART (3–11). Recently, our lab and others reported an aberrant accumulation of virus-infected Tfh cells in the lymph nodes (LNs) and rectal mucosa of SIV-infected rhesus macaques with high viral load (10, 12, 13). Interestingly, HIV controllers who develop robust antiviral CD8 T cell responses, the virus appears to preferentially persist in these LN follicles (12–15). Therefore, the lymphoid tissue serves as the principal reservoir site of infected cells, and its BCF compartment harboring reservoirs represents a unique site where cytolytic cells such as CD8^+^ T cells and NK cells have limited access (14, 16). Thus, there is a need to identify immune mechanisms capable of eliminating infected cells from these lymphoid structures.

NK cells are a component of the innate immune system that play a central role in host defense against viral infections (17–19). These cells kill infected target cells via targeted release of cytolytic granules and release of antiviral cytokines (20). Indeed, NK cells influence viral control during HIV/SIV infection putatively via direct cytotoxicity of infected cells, antibody-dependent cellular cytotoxicity (ADCC), or the release of beta chemokines (21–23). NK cells have been associated with slower disease progression (21, 24) and inhibition of mother-to-child transmission of HIV (24–26). Furthermore, elevated NK cell frequencies correlate with protection from virus acquisition in healthy seronegative individuals (27–30). Therefore, understanding the nature and role of NK cells in lymphoid tissues under chronic HIV/SIV infection is critical.

Unfortunately, studies in SIV-infected macaques and humans infected with HIV indicate that NK cells typically exhibit a limited capacity to migrate into the BCFs and germinal centers (GCs) of secondary lymphoid tissue (14, 31–35). In contrast, accumulation of NK cells within BCFs is a key parameter in SIV control in the nonpathogenic African green monkey model (36–38). Here, we observed a strong induction of BCF-homing CXCR5^+^ NK cells in the LNs of macaques during chronic SHIV infection, the magnitude of which was associated with a superior viral control. These CXCR5^+^ NK cells showed unique gene and protein expression profiles associated with elevated functional capacity and cytokine signaling. Consistent with this observation, treatment with the cytokines IL-12 and IL-15 enhanced the generation and proliferation of highly functional CXCR5^+^ NK cells. Thus, CXCR5^+^ NK cells represent a unique subset of NK cells with the potential to home to BCFs and exert superior antiviral functionality. These findings have implications for curative HIV strategies.

## Results

### CXCR5^+^ NK cells expand in LNs after chronic SHIV infection.

**Figure 1:**
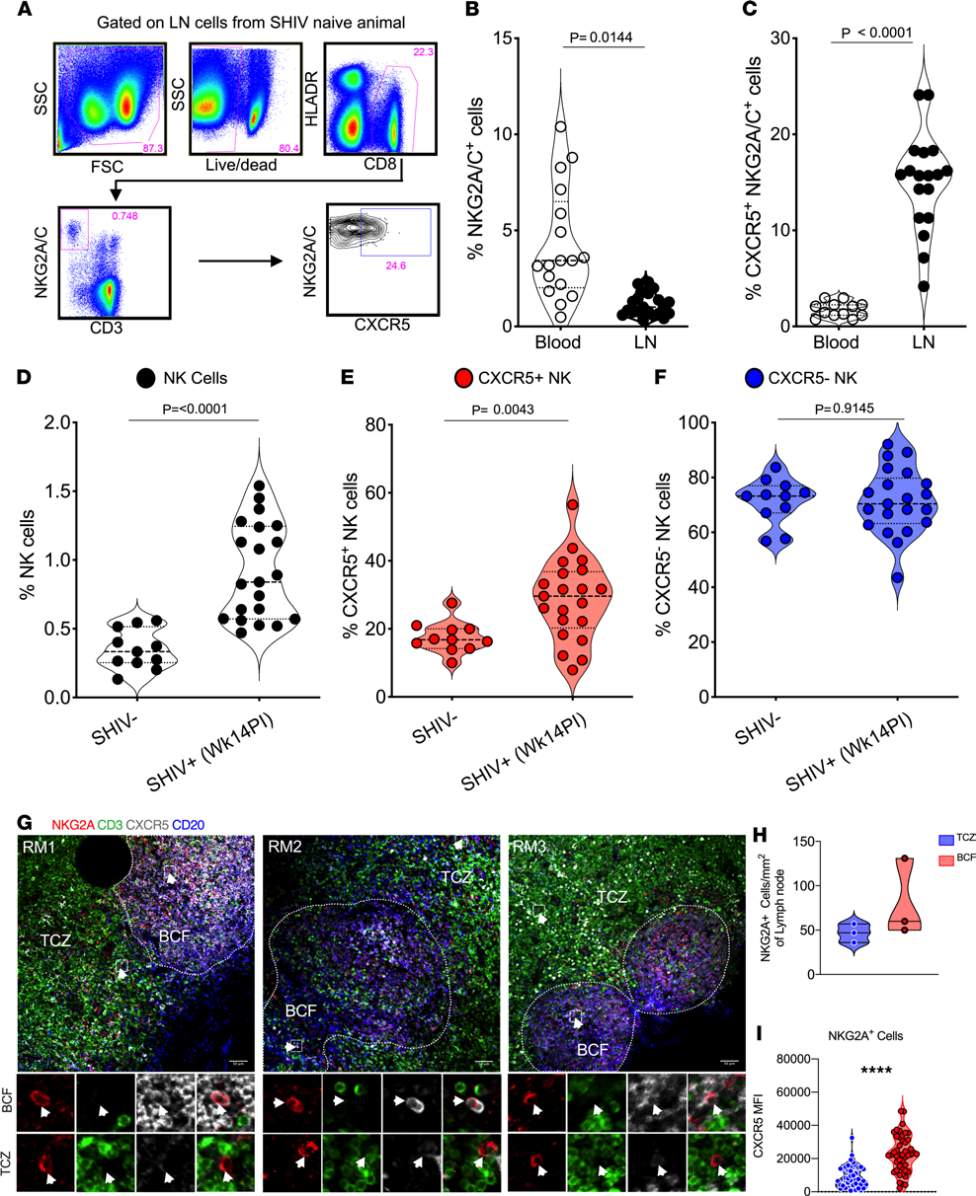
Accumulation of CXCR5^+^ NK cells in B cell follicles during SHIV infection. (**A**) Gating strategy for CXCR5-expressing NK cells is shown. Frequency of NK cells (**B**) and CXCR5-expressing NK cells (**C**) in blood (*n* = 17) and lymph nodes (LNs) (*n* = 17) of uninfected macaques. Proportions of total NK cells (**D**), CXCR5^+^ (**E**), and CXCR5^–^ NK cells (**F**) at preinfection day 0 (*n* = 11) and week 14 of SHIV infection (*n* = 21). (**G–I**) IHC images from 3 representative SHIV-infected (14 weeks after infection) macaques showing CD20 (blue), CD3 (green), NKG2A (red), and CXCR5 (gray) staining of LN sections (**G**). Mean number of NK cells per mm square area of B cell follicle (BCF) and T cell zone (TCZ) compartments of LN sections from 3 macaques (**H**). MFI ± SEM of CXCR5 on NKG2A^+^ cells (*n* = 46) in BCF and TCZ (**I**). *P* value was calculated using Mann-Whitney test. Scale bar: 50 μm. Wilcoxon’s matched-pairs signed rank test was used to compare the frequencies of CXCR5^+^ NK cells between blood and LN. Arrows indicate NK cells. ****P < 0.0001.

In order to elucidate the distribution and function of NK cells in rhesus macaques, we stained cells for NKG2A expression in the blood and LN compartments (Figure 1A). The proportion of NK cells was significantly lower in LNs (*P* = 0.01) relative to the blood compartment (Figure 1B), however, the fraction of NK cells expressing CXCR5 was significantly (*P* < 0.0001) higher (5-fold) in LNs compared with the blood compartment (Figure 1C). We performed additional gating to ensure the NK cell population did not include B cells or NK-like B (IgM^+^) cells (Supplemental Figure 1, A and B; supplemental material available online with this article; https://doi.org/10.1172/jci.insight.155601DS1). After chronic SHIV infection (week 14 after infection; see viral load levels in Supplemental Table 1), the frequency of NK cells (Figure 1D) and proportion expressing CXCR5 in the LNs increased significantly from baseline in contrast to the frequency of CXCR5^–^ NK cells (Figure 1, E and F). This enhancement of CXCR5 expression was not seen in the blood after chronic SHIV infection (Supplemental Figure 1C). To ascertain the localization and distribution of NK cells in the B cell follicle (BCF) and T cell zone (TCZ) of the LNs in SHIV-infected rhesus macaques, we performed IHC analysis. We observed CD3^–^NKG2A^+^ NK cells within the BCF as well as the TCZ of the lymphoid tissue (Figure 1G). Quantification of these cells revealed enhanced localization in the BCF (~80 NKG2A^+^ cells/mm^2^) compared with the TCZ (~47 NKG2A^+^ cells/mm^2^), though not significant (Figure 1H). We further analyzed MFI of the CXCR5 on NKG2A^+^ cells inside the BCF and TCZ and found significantly higher CXCR5 MFI (*P* = 0.0001) in the BCF compared with the TCZ (Figure 1I). These data showed that chronic infection was associated with increased CXCR5 expression and increased localization of NK cells within BCFs, which are major sites of viral reservoir persistence.

### CXCR5^+^ NK cells are phenotypically and functionally distinct from CXCR5^–^ NK cells.

**Figure 2:**
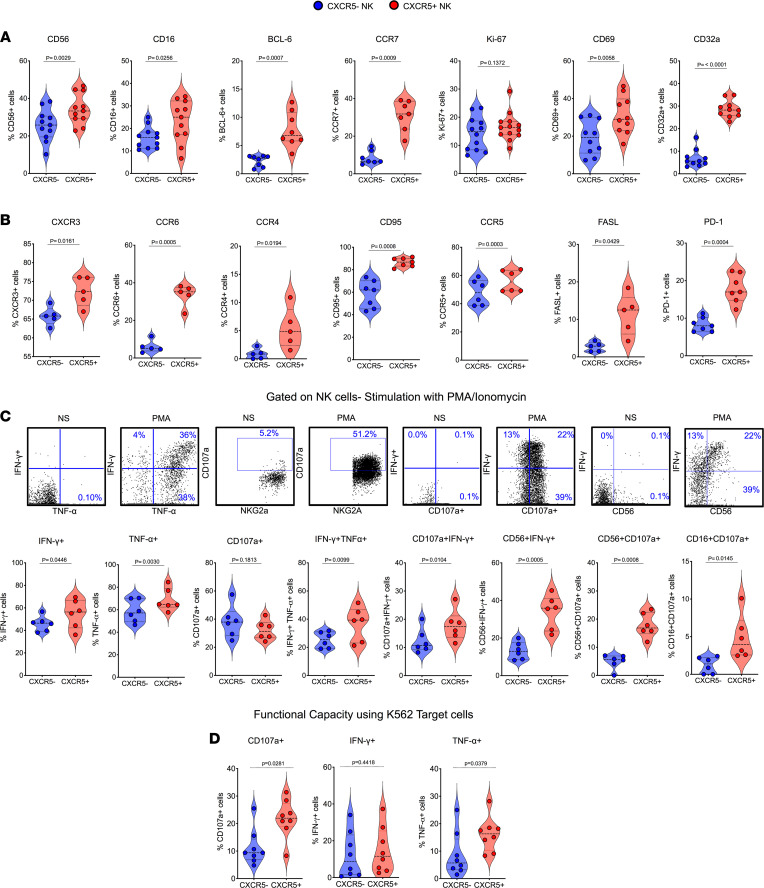
Follicular CXCR5^+^ NK cells display activated phenotype and heightened functionality. (**A**) Violin plots showing the expression of different indicated markers (CD56, CD16, BCL-6, CCR7, Ki-67, CD69, CD32a) on CXCR5^+^ and CXCR5^–^ NK cells; data shown for week 14 after SHIV infection (*n* = 11). (**B**) Expression of chemokine receptors (CXCR3, CCR6, CCR4) and CD95, CCR5, FASL, PD-1 on CXCR5^+^ and CXCR5^–^ NK cells (*n* = 6). (**C**) Cytokine expression and degranulation (CD107a^+^ staining) profiles of CXCR5^+^ and CXCR5^–^ NK cells after 6 hours of ex vivo culture in presence (PMA) or absence (NS) of PMA and ionomycin determined by intracellular cytokine staining and flow cytometry (*n* = 6). (**D**) Similar assessment of functionality of CXCR5^+^ and CXCR5^–^ NK cells after 6 hours of coculture with MHC class I–devoid K562 target cells (*n* = 8). Wilcoxon’s matched-pairs signed rank test was used to compare the frequencies of CXCR5^+^ and CXCR5^–^ NK cells in the lymph nodes.

Human and nonhuman primate Tfh cells as well as follicular CD8^+^ T cells are characterized by high expression of CXCR5 and BCL-6 but lower expression of CCR7 (13, 34, 35, 39). In a similar fashion, CXCR5^+^ NK cells expressed higher levels of BCL-6 compared with CXCR5^–^ NK cells (Figure 2A). Of note, CXCR5 expression was higher on NK cells than total LN CD8^+^ T cells in SHIV-infected rhesus macaques (Supplemental Figure 1D). These animals displayed typical viral kinetics after chronic SHIV infection as indicated by plasma viral load (week 14 after infection) (Supplemental Figure 1E). Interestingly, and unlike follicular CD4^+^ T cells, CXCR5^+^ NK cells expressed higher levels of CCR7 compared with their CXCR5^–^ counterparts (Figure 2A). CXCR5^+^ NK cells exhibited a strong activation profile with elevated expression of CD56, CD69 (Figure 2A), CD95, CCR5, FASL, and PD-1 (Figure 2B); see details of antibodies/clones used in (Supplemental Table 2). Furthermore, CXCR5^+^ NK cells expressed higher levels of the Fc receptors FcγRIII (CD16) and FcγRIIa (CD32a), which might indicate a higher capacity of these cells to engage in antibody-dependent effector functions. Because we observed higher expression of FCγ receptors on the CXCR5^+^ NK cells, which is the marker of enhanced antibody-dependent function, we performed a CD16 cross-linking experiment with the LN cells from the SHIV-infected macaques to test their cytotoxic potential. Interestingly, CXCR5^+^ NK cells showed significantly higher degranulation indicated by CD107a (*P* = 0.03) expressing significantly higher TNF-α^+^ (*P* = 0.01) and IFN-γ^+^ (*P* = 0.002) cytokines compared with the CXCR5^–^ NK cells (Supplemental Figure 2A). These CXCR5^+^ NK cells expressed a significantly higher proportion of CD56 that showed enhanced cytokine expression (Supplemental Figure 2B). In addition to their elevated expression of CXCR5 and CCR7, these CXCR5^+^ NK cells exhibited heightened expression of additional chemokine receptors, including CXC3, CCR6, and CCR4 (Figure 2B), revealing a generally enhanced capacity for tissue migration of this subset of NK cells.

To compare the functional quality of LN CXCR5^+^ and CXCR5^–^ NK cells, we investigated their responses to broad (PMA/ionomycin) and NK cell–specific (K562 target cell) stimulation. Compared with the CXCR5^–^ subset, CXCR5^+^ NK cells exhibited enhanced capacity to express IFN-γ^+^ (*P* = 0.04) and TNF-α^+^ (*P* = 0.003) cytokines after stimulation with PMA/ionomycin (Figure 2C), which showed higher degranulation (*P* = 0.01) compared with the CXCR5^+^ NK cells in SHIV-naive macaques (Supplemental Figure 2C). Encouragingly, CXCR5^+^ NK cells showed superior polyfunctionality as indicated by increased capability to degranulate (CD107a^+^) concomitant with the cytokine expression compared with the CXCR5^–^ NK cells (Figure 2C). Similarly, we observed enhanced degranulation CD107a^+^ (*P* = 0.028) and TNF-α^+^ (*P* = 0.03) expression after coculture with MHC devoid K562 cells (Figure 2D) compared with the CXCR5^–^ NK cell subset and superior TNF-α^+^ cytokine expression by the CXCR5^+^ NK cells in the chronic SHIV condition compared with CXCR5^+^ NK cells from SHIV-naive macaques (Supplemental Figure 2D). Collectively, these data suggested that CXCR5^+^ NK cells are phenotypically distinct and possess superior functional capability compared with their CXCR5^–^ counterparts.

### CXCR5^+^ NK cells exhibit transcriptomic features associated with lymphoid progenitors and are poised for cytolytic function.

**Figure 3:**
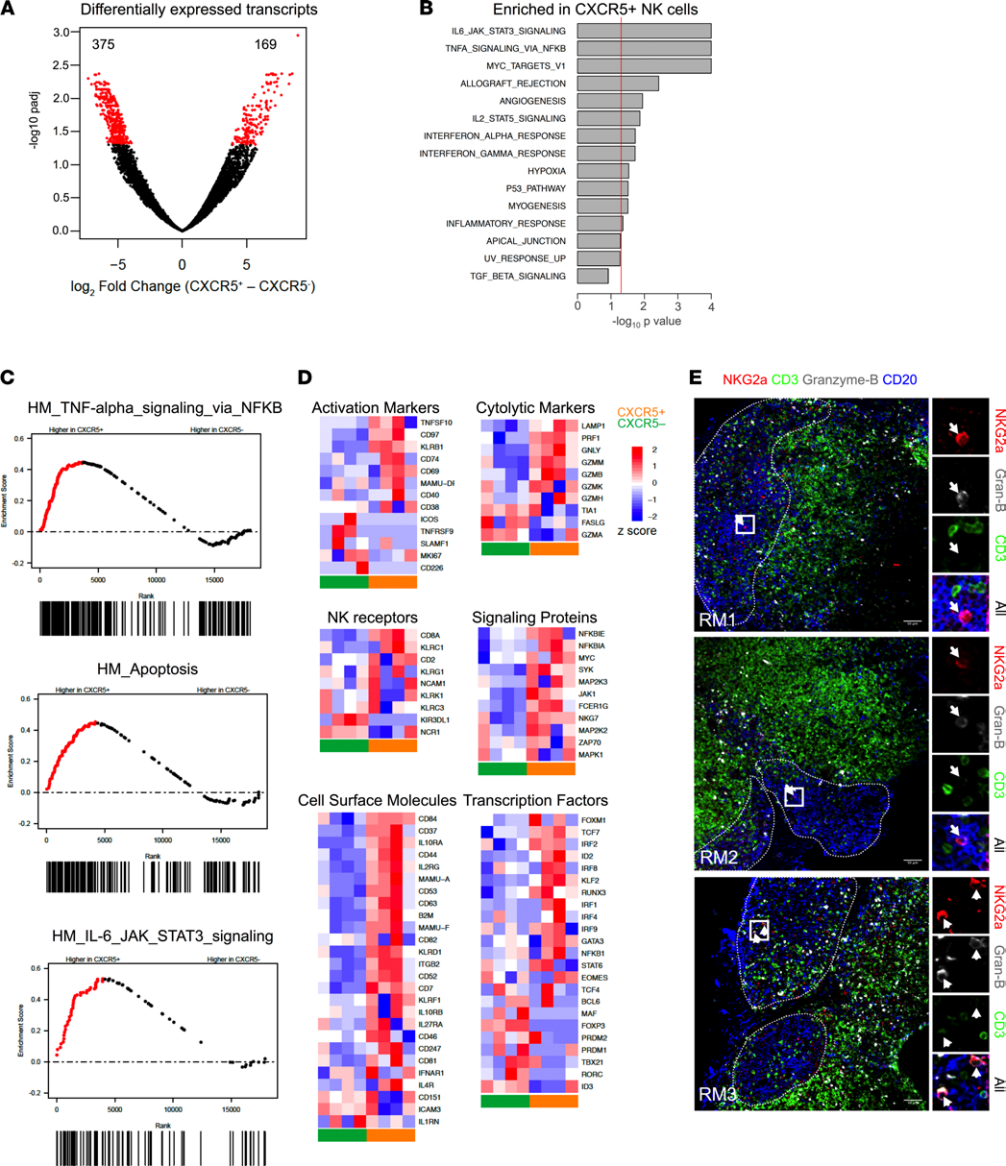
CXCR5^+^ NK cells are transcriptionally distinct from CXCR5^–^ NK cells during chronic SHIV infection. (**A**) Purified NK cells from lymph nodes (LNs) at week 14 after infection were RNA sequenced, and volcano plots were analyzed for differentially expressed transcripts (*n* = 4). (**B**) Normalized enrichment scores for upregulated gene sets in CXCR5^+^ NK cells are depicted. Dashed line indicates normalized enrichment score cutoff of greater than 135 with FDR of less than 0.2. (**C**) GSEA plots comparing CXCR5^+^ and CXCR5^–^ NK cells. (**D**) Global gene expression analysis showing gene expression profile for CXCR5^+^ and CXCR5^–^ NK cells. The color intensity for heatmaps represents *Z* score of differential expression by CXCR5^+^ versus CXCR5^–^ NK cells, calculated as described in Methods. (**E**) IHC images showing CD20 (blue), CD3 (green), NKG2A (red), and granzyme B (gray) staining of LN sections of SHIV-infected macaques. Scale bar: 50 μm. Arrow indicates NK cells.

To elucidate the cellular and functional differences between the CXCR5^+^ and CXCR5^–^ NK cell subsets during chronic SHIV infection, we performed global gene expression RNA-Seq analysis on subsets of cells sorted from LNs of 4 chronically SHIV-infected rhesus macaques (Figure 3). Comparing CXCR5^+^ with CXCR5^–^ NK cells, about 544 genes were differentially expressed (Figure 3A), with a greater number upregulated in the CXCR5^+^ subset. Gene set enrichment analysis (GSEA) revealed that genes involved in IL-6, JAK-STAT3, TNF-α, and MYC signaling were enriched in CXCR5^+^ NK cells (Figure 3, B and C, and Supplemental Figure 3). Importantly, these signaling pathways are also shown to be enriched in GC-Tfh cells from macaques and humans (4, 40). CXCR5^+^ NK cells exhibited greater expression of activation markers like *CD44*, *CD84*, *KLRB1*, *TNFSF10*, *CD97*, and *CD69* (Figure 3D). The CXCR5^+^ subset of NK cells was also characterized by an effector-like phenotype, including elevated expression of *CD8A*, *KLRC1*, and *CD2*. Expression of genes involved in granule-mediated cytotoxicity, such as perforin (*PRF1*), granulysin (*GNLY*), and various granzymes (*GZMM*, *GZMB*, *GZMK*, *GZMH*), were also higher in CXCR5^+^ NK cells compared with CXCR5^–^ NK cells (Figure 3D). CXCR5^+^ NK cells expressed higher levels of transcripts encoding the cytokine receptors *IL10RA*, *IL2RG*, *IL10RB*, *IL27RA*, *IFNAR1*, and *IL4R*. Likewise, CXCR5^+^ NK cells also showed higher expression of *NFKB*, *MAP2K3*, *SYK*, *JAK1*, *NKG7*, and *ZAP70,* which are involved in cellular activation pathways (41). In contrast to the CXCR5^–^ NK cells, CXCR5^+^ NK cells exhibited elevated expression of genes associated with the lymphoid progenitors, including *TCF7*, *MYC*, *RUNX3*, *NFKB1*, *SELL*, *CD127*, *XCL1*, *CD27*, and *CCR7*, whereas CXCR5^–^ NK cells were characterized by higher expression of classic NK cell factors like *PRDM1*, *MAF*, and *TBX2,* which are associated with non-lymphoid localization programs (41). Moreover, we directly observed the presence of GrzB^+^ NKG2A^+^ cells inside BCFs (Figure 3E). Collectively, these results demonstrated that CXCR5^+^ NK cells possess a unique gene expression pattern that is distinct compared with CXCR5^–^ NK cells and show features associated with effector functions, in addition to having lymphoid progenitor–like characteristics.

### CXCR5^+^ NK cells are associated with reduced plasma viral RNA levels and GC-Tfh cell frequencies.

**Figure 4:**
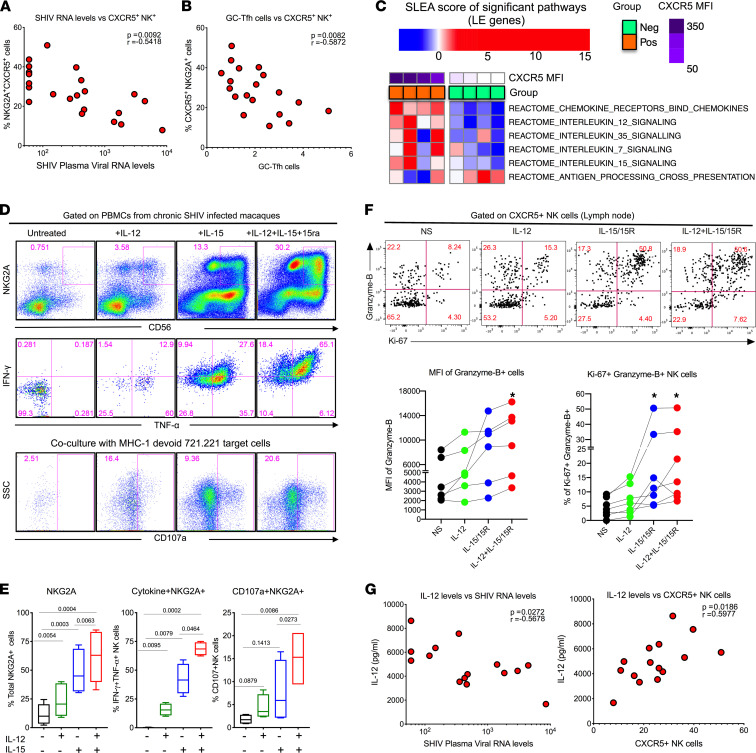
Combination IL-12 and IL-15 cytokine treatment improves proliferative and cytotoxic capacity of CXCR5^+^ NK cells. (**A** and **B**) Pearson’s correlation between CXCR5^+^ NK cells (in LNs) and SHIV plasma viral RNA (*n* = 21) (**A**) and GC-Tfh (**B**) (*n* = 19). (**C**) GSEA revealed enriched cytokine signaling in CXCR5^+^ NK cells relative to CXCR5^–^ counterparts. (**D** and **E**) Effect of in vitro stimulation with combination of IL-12 and IL-15 cytokines for 72 hours on NK cell functional properties (*n* = 4). (**E**) Frequency of NKG2A^+^ cells, cytokine expression, and degranulation. (**F**) Proliferation and granzyme expression on CXCR5^+^ NK cells in LNs are shown; data for 6 animals are indicated. (**G**) Correlation between plasma levels of IL-12 cytokines and SHIV RNA levels (*n* = 15). Correlation between plasma levels of IL-12 cytokines and CXCR5^+^ NK cells. To correct for multiple correlations, we performed Bonferroni’s correction. Assuming the overall significance level to be 0.05, the significance threshold for individual correction will be 0.05/2 = 0.025. Under this significance threshold, both comparisons are considered significant. For *P* value, Mann-Whitney test was used for in vitro analysis. Pearson’s correlation was used for correlation analysis.

To understand the relationship between LN CXCR5^+^ NK cells and viral control, we analyzed the correlation between frequencies of CXCR5^+^ NK cells and plasma viral load. In addition, we also analyzed the correlation between the LN CXCR5^+^ NK cells and the GC-Tfh cells (potential viral reservoirs within BCFs) in macaques after pathogenic SHIV infection. Notably, the frequency of CXCR5^+^ NK cells displayed a strong inverse correlation (*r* = –0.54) with the plasma viral RNA levels (Figure 4A). Similar to the correlation with the viral SHIV RNA level, the frequency of LN CXCR5^+^ NK cells was found to be inversely associated (*r* = –0.59) with the frequency of the GC-Tfh cells (Figure 4B). These results strongly suggested a role of CXCR5^+^ NK cells in controlling infected cells within BCFs.

### Cytokines IL-12 and IL-15 enhance the functional quality of CXCR5^+^ NK cells in vitro.

In order to investigate potential pathways for increasing the CXCR5^+^ expression on the NK cells as a means to enhance BCF localization of NK cells and mediate viral control by eliminating reservoirs, we evaluated GSEA probing alterations in major cytokine/chemokine gene sets (obtained from Reactome database — see Supplemental Table 3) in sorted CXCR5^+^ and CXCR5^–^ NK cell subsets from LNs. We observed 6 gene sets were significantly associated (*P* < 0.05; Supplemental Table 4) with the expression level of CXCR5 (MFI measured by flow cytometry) on NK cells. Leading-edge genes that comprised these gene sets were aggregated per sample using sample level enrichment analysis (SLEA) (42), and heatmaps were generated to visualize alterations in chemokine/cytokine signaling cascades across NK cell subsets (Figure 4C). CXCR5^+^ NK cells were enriched in genes encoding for chemokine receptors (characterized by higher levels of *CCR6* and *CCR7*) and the IL-15 signaling cascade (characterized by higher levels of *IL15RA*, *IL2RG*, *JAK1*, *STAT5A*) (Supplemental Tables 3 and 4). These data are in line with previous findings (36) that demonstrate CXCR5^+^ NK cells are regulated by IL-15 in African green monkeys (AGMs) and complement the fact that BCFs are enriched for IL-15 (36, 43). In addition to the increase in IL-15 signaling, we observed that NK cells with higher CXCR5 levels were significantly enriched in IL-12 family signaling (characterized by higher levels of *IL12RB1/2* and *JAK1/2*) (Figure 4C and Supplemental Tables 3 and 4). Therefore, in order to characterize the relevance of IL-12 and IL-15 cytokines signaling to CXCR5^+^ NK cell biology, we treated blood mononuclear cells from SHIV-infected rhesus macaques with IL-12, IL-15/IL-15Ra, or a combination of these cytokines for 3 days. IL-15/IL-15Ra treatment markedly increased the frequency and function (degranulation in response to MHC class 1–devoid 721.221 target cells for 6 hours) of NK cells (Figure 4, D and E). IL-12 treatment alone showed only marginal improvement of NK cell frequency and function. However, the combination of IL-12 and IL-15/IL-15Ra showed a profound increase in the frequency and functional properties of NK cells that was greater than the effect of either single cytokine treatment. Next, we investigated the effect of cytokine treatment on the effector function of LN-derived NK cells in vitro. Both IL-12 and IL-15 synergistically enhanced granzyme B^+^ Ki67^+^ expression on CXCR5^+^ NK cells in LNs (Figure 4F). Next, we measured the levels of plasma cytokines in these animals before and after SHIV infection (week 14 after SHIV). The levels of IL-12 displayed a marked increase**,** but the levels of IL-15 did not change (Supplemental Figure 4, A and B). The IL-12 levels displayed negative correlation with viral load and direct correlation with the frequency of CXCR5^+^ NK cells, but IL-15 levels were not correlated (Figure 4G and Supplemental Figure 4, C and D). Taken together, these data suggested that combining IL-12 and IL-15/IL-15Ra cytokine treatment synergized in potentiating the functional properties of the NK cells, including follicular homing and enhanced effector function, for enhanced antiviral effect during chronic SHIV infection.

## Discussion

In this study, we investigated the dynamics and functional properties of CXCR5-expressing NK cells having BCF homing potential during chronic SHIV infection in rhesus macaques. Our findings revealed a rapid expansion of a subset of highly functional CXCR5^+^ NK cells with a potential to migrate to the BCFs in the LNs and carrying a unique phenotype and functional characteristics in SHIV-infected rhesus macaques. Importantly, the expansion of CXCR5^+^ NK cells in the LNs was associated with better control of pathogenic SHIV infection. Our results also showed that the frequency of CXCR5^+^ NK cells was negatively associated with the GC-Tfh cells, which may imply that CXCR5^+^ NK cells could restrict expansion of Tfh cells in chronic SHIV infection. NK cells have previously been shown to interact with other cell populations such as Tfh cells and B cells (44–46). Also, it is not clear how IFN-γ secreted by NK cells affected Tfh functions in chronic SHIV-infected macaques. Given that IFN-γ is known to augment CD4^+^ Th type I function, it is not clear if lower Tfh frequencies associated with higher CXCR5^+^ NK cells had any better functional quality. Furthermore, the association of NK cell–mediated killing of SHIV-infected T cells may not be limited to the BCFs that would be contributing to the plasma viremia. In fact, it has been shown that T cells outside of BCFs also contribute to productive infection and reservoir formation (47). In addition, HIV/SIV reservoirs as far as the brain can also contribute to plasma viremia (48). These finding warrant detailed investigation of the association of NK cells and viral RNA^+^ (vRNA^+^) cells in different tissue compartments. Also, the mechanisms by which these cells are able to restrict Tfh expansion require further investigation. In the current study, several lines of evidence suggested that CXCR5^+^ NK cells are highly cytotoxic, as they expressed perforin, granzymes, NKG7, granulysin, and LAMP-1. In addition, these cells expressed higher levels of the Fcγ receptors CD16 and CD32, suggesting these cells possess higher antibody-dependent effector functionality compared with CXCR5^–^ NK cells. Cross-linking of CD16 on CXCR5^+^ NK cells expressed higher levels of degranulation and cytokine production compared with CXCR5^–^ NK cells, suggesting that CXCR5^+^ NK cells have higher cytotoxic potential. Furthermore, CXCR5^+^ NK cells made functional cytokines with mitogen stimulation and with a coculture experiment with MHC class 1–devoid K562 target cells. A direct demonstration of the killing potential of CXCR5^+^ NK cells will be key to know its precise functional nature. Our attempts to perform a CXCR5^+^ NK cells versus T-cell–killing assay ex vivo were not successful because this assay requires a large number of sorted CXCR5^+^ NK and GC-Tfh cells.

It is interesting to note that CXCR5^+^ NK cells have been shown to express transcripts/proteins associated with lymphoid progenitor markers (41). Consistently, TCF-1 and MYC marker expression indicate that CXCR5^+^ NK cells express gene signatures associated with LN localization, whereas the CXCR5^–^ NK cells express PRDM1-ZEB2 phenotypes and non-lymphoid homing gene signature programs (41). This observation raises the possibility that cytokines and signals that shape Th differentiation programs may also play a role in the differentiation of CXCR5^+^ NK cells. Accordingly, we observe that aside from IL-15 (the prototypic cytokine known to orchestrate cytotoxic CD8 and NK cell responses), genes involved with IL-12 signaling (the primary driver of Th 1 response) were enriched in CXCR5^+^ NK cells. It should be noted that the enrichment in these cascades was characterized by expression of surface receptors (higher *IL-15RA, IL-2RG, IL-12RB1/2*) and of master transcriptional regulators, such as of IL-12 and IL-15 signaling (*STAT5A*, *STAT4*, *JAK1/2*). These cytokines are known to activate NK cell functions in human secondary lymphoid organs (49). Data characterizing the implication of the enrichment of these cascades in NK cells during SHIV infection in lymphoid tissues remains limited. Understanding the mechanisms underlying the induction of CXCR5^+^ NK cells by therapeutic intervention during infection — or better, before vaccination — is critical for developing immune-based strategies that can substantially reduce the HIV reservoir and generate effective control of viral replication in the absence of ART.

Our in vitro studies showed that cytokines IL-12 and IL-15 might play an important role in inducing CXCR5^+^ NK cells with superior functional and LN homing properties. Previous studies showed that IL-15 treatment induced these cells in vivo (50). Consistently, we found that IL-15 in combination with IL-12, which has been shown to promote activation of NK cells with higher CD56 expression, also enhanced granzyme B expression in CXCR5^+^ NK cells. In addition, we also found that levels of IL-12 displayed negative correlation with plasma viral load and positive correlation with the CXCR5^+^ NK cells. Further investigation into early immune signatures will help to address what mechanisms influence the in vivo generation of these CXCR5^+^ NK cells. Our in vitro studies suggest that IL-12 in combination with IL-15 synergistically acts to activate induction of cytolytic NK cells with BCF-homing potential (CXCR5^+^ NK cells). Furthermore, our in vitro cytokine treatment results suggested that the IL-12– and IL-15–mediated pathway could be modulated in vivo to augment the induction of CXCR5^+^ NK cells. Previous studies have shown that levels of IL-12 and IL-15 decrease significantly after SIV and HIV infections (51, 52) and both cytokines (IL-12 and IL-15) increase the degranulation capacity of the NK cells during HIV infection (53). Moreover, IL-15 super-agonism has also been shown to promote SIV-specific CD8^+^ T cells and NK cells into BCFs (50, 54–56). Therefore, further investigation into early immune signatures will help to address mechanisms influencing the in vivo generation of these CXCR5^+^ antiviral NK cells and their specific contribution in viral control.

The recently described follicular CXCR5^+^ NK cells from chronic lymphocytic choriomeningitis virus (LCMV) and nonpathogenic SIV infections (17, 36, 44) have been shown to express the transcription factor BCL-6, similar to what we have observed in our CXCR5^+^ NK cells, and are selectively present in the BCFs. These cells were found to negatively associate with the GC-Tfh cells. In addition, these cells expressed the TCF-1 transcription factor and behaved as lymphoid progenitors as described before (41). These observations imply that a similar mechanism may induce these CXCR5^+^ NK cells, highlighting the importance of harnessing the mechanisms underlying the induction of CXCR5^+^ NK cells, to design strategies that will induce potent CXCR5^+^ NK cells with superior cytolytic functions, thereby bringing effective viral control in the absence of ART. In addition, the magnitude of CXCR5^+^ NK cells observed in current study wherein the rhesus macaques were infected with SHIV was comparable with the magnitude shown in the LNs of the SIV-infected AGMs (36). Interestingly, the NK cell frequency was higher compared with what was observed in the SIV-infected cynomolgus macaques in the same study. We speculate this difference could be contributed by variations in both studies, such as the kind of virus used for infection (SHIV versus SIV), kinetics of viral pathogenesis, virus dose, route of infection (intrarectal versus intravenous), weeks after infection (14 versus 34 weeks), and nonhuman primate species (rhesus versus cynomolgus).

Our study has limitations. We have not shown direct NK cell–mediated killing of SHIV-infected cells in LNs or other tissue compartments. Our in vitro experiments have pointed to CXCR5^+^ NK cells having more cytolytic potential, and their killing properties could have been tested in vitro. In addition, the spatial and temporal association of cytolytic NK cells with reference to SHIV-infected cells could have been defined in more detail. However, given a lack of samples, we could not perform a thorough investigation. In the current study, we investigated NK cell properties and their association with viral control during chronic SHIV infection. It is also important to study the contribution of NK cells in SHIV control in early stages of the infection in other anatomical sites as well. These limitations warrant further detailed investigation.

In conclusion, our results showed that chronic SHIV infection significantly induced CXCR5^+^ NK cells in LNs, these cells carried superior functional properties, and the cells were found localizing into the BCFs of chronically infected rhesus macaques. Importantly, we showed that these NK cells associated strongly with a decrease in the pathogenic SHIV viral load, suggestive of better viral control. In addition, we showed that in vitro IL-12 and IL-15 cytokine combination treatment synergistically enhanced the functional quality of CXCR5^+^ NK cells and their association with CXCR5-expressing NK cells. Inducing these cells by therapeutic approaches provides a unique opportunity to develop and optimize strategies that can successfully target and reduce the viral reservoir in lymphoid tissues. Altogether our data highlight the significance of inducing CXCR5^+^ NK cells by therapeutic or vaccination strategy to enhance control of chronic HIV infection. Our findings may have important implications for HIV cure strategies.

## Methods

### Ethics statement.

All animal experimentations were conducted at the Yerkes National Primate Research Center (YNPRC), which is accredited by American Association of Accreditation of Laboratory Animal Care International, following guidelines established by the Animal Welfare Act and the NIH for housing and care of laboratory animals. Blood and tissue collections were obtained under anesthesia. Rhesus macaques were fed standard monkey chow (Jumbo Monkey Diet 5037, Purina Mills) supplemented with fresh fruit or vegetables daily. Consumption was monitored and adjustments were made as necessary depending on sex, age, and weight. SHIV-infected rhesus macaques were singly caged but had visual, auditory, and olfactory contact with at least one social partner, permitting the expression of noncontact social behavior. The YNPRC enrichment plan employs several general categories of enrichment. This study was performed in strict accordance with the recommendations in the NIH *Guide for the Care and Use of Laboratory Animals* (National Academies Press, 2011), a national set of guidelines in the United States, and to international recommendations detailed in the Weatherall Report (2006). This work received prior approval by the IACUC of Emory University. Appropriate procedures were performed to ensure that potential distress, pain, discomfort, and/or injury was limited to that unavoidable in the conduct of the research plan. Ketamine (10 mg/kg) and/or Telazol (4 mg/kg) were used for collection of blood and tissues, and analgesics were used when determined appropriate by veterinary medical staff.

### Animals.

Indian adult rhesus macaques obtained from the YNPRC breeding colony were cared for under the guidelines established by the Animal Welfare Act and the NIH *Guide for the Care and Use of Laboratory Animals* (National Academies Press, 2011) using protocols approved by the Emory University IACUC. All rhesus macaques were infected with the SHIV1157ipd3N4 stock (1:400) dilution intrarectally. The SHIV-uninfected LN samples were collected before SHIV infection and served as SHIV-naive samples. Some animals were positive for Mamu A*01 allele, and all animals were negative for Mamu B08 and Mamu B17. All animals were free of simian retrovirus type D or simian T-lymphotropic virus type 1. LN tissue samples from 22 Indian rhesus macaques were analyzed in this study. Animals were infected intrarectally, and chronically SHIV-infected animals were euthanized at week 14 after SHIV infection; see details of respective plasma viral load kinetics shown in Supplemental Figure 1E.

### Macaque samples.

Whole blood and LN mononuclear cells were isolated using standard isolation protocols. PBMCs were isolated by density gradient centrifugation layered over 100% Ficoll. LN mononuclear cells were isolated by mechanical disruption. Contaminating RBCs were lysed using an ACK lysis buffer (Gibco, A1049201). Cell aliquots were immediately cryopreserved in 90% FBS–10% dimethyl sulfoxide (Sigma-Aldrich) and stored in liquid nitrogen vapor.

### Tissue collection and processing.

Axillary and inguinal lymph node (PLN) tissues were collected from humanely euthanized macaques at the necropsy time points. Preinfection PLN biopsy specimens were taken from naive animals; for the acute infection, biopsies were collected on day 21 after infection. PBMCs were isolated by density gradient centrifugation of EDTA-treated blood over lymphocyte separation medium (MP Biomedicals), followed by lysis of RBCs using a hypotonic ammonium chloride solution.

### Cytokine and cytokine treatments.

Recombinant macaque IL-12, IL-15, and IL-15Rα-IgFc were produced by the Resource for Nonhuman Primate Immune Reagents (New Iberia Research Center, University of Louisiana at Lafayette).

### Phenotyping.

Mononuclear cells were processed from the blood and LNs and stained with LIVE/DEAD Near-IR Dead Cell at room temperature for 15 minutes in PBS to stain for dead cells. Cells were then washed with FACS wash, stained on the surface using antibodies specific to respective cell markers, and then treated with 1× BD Biosciences FACS lysing solution for 10 minutes at room temperature, permeabilized with 1× BD Biosciences permeabilizing solution for 10 minutes at room temperature, washed with FACS wash, stained intracellularly using antibodies specific to the respective intracellular markers, washed 2× with FACS wash, and assessed by flow cytometry, as described previously (57, 58).

### Antibodies and flow cytometry.

All antibodies were purchased from BD Biosciences unless specified otherwise, and clone information is given in parentheses. Antibodies to the following antigens were used in this study: LIVE/DEAD fixable Near-IR Dead Cell stain (Invitrogen), CD3 (clone SP34.2), CCR7 (clone 150503; R&D Systems), CD8α (clone T8/7Pt-3F9, Nonhuman Primate Reagent Resource Program), CD8α (clone SK1), CD16 (clone 3G8), CD56 (clone NCAM16.2), NKG2A-PE (clone Z199; Beckman-Coulter), CD20 (clone 2H7), HLA-DR–Percp (G46-6), CXCR3 (clone IC6), CCR6 (clone 11A9), CCR7 (clone 150503, R&D Systems), CCR4 (clone 1G1), CD32a (clone FL18.26), CD69 (clone FN50), Ki67 (clone B56), perforin (clone Mab-Pf344), granzyme B (GB-11), BCL-6 (clone K112-91), IFN-γ (clone B27), TNF-α (Mab11), CD107a (clone H4A3). See Supplemental Table 2 for more details.

### Intracellular cytokine staining.

Mononuclear cells were incubated with PMA (100 ng/mL) and ionomycin (1 μg/mL) or cultured in RPMI 1640 containing 10% FBS (R10) alone. For all samples, anti-CD107a (FITC, clone H4A3) was included at a concentration of 20 μL/mL, and Golgi Plug (brefeldin A) and Golgi Stop (monensin) were included at 6 μg/mL. Samples were incubated for 6 hours at 37°C in 5% CO_2_ and then permeabilized using Fix & Perm reagents (Invitrogen) and stained intracellularly with anti–IFN-γ (PE-Cy7 conjugate, clone B27; Invitrogen), and anti–TNF-α (Alexa Fluor 700 conjugate, clone Mab11). At the end of stimulation, cells were washed once with FACS wash (PBS containing 2% [vol/vol] FBS and 0.25% of sodium azide) and surface stained with anti-CD3, anti-NKG2A, anti-CD8, anti-CD4, and LIVE/DEAD Near-IR Dead Cell stain (Life Technologies) at room temperature for 30 minutes. Cells were then fixed with Cytofix/Cytoperm (BD Pharmingen) for 20 minutes at 4°C and washed with Perm wash (BD Pharmingen). Cells were then incubated for 30 minutes at 4°C with antibodies specific to IFN-γ and TNF-α, washed once with Perm wash and once with FACS wash, and resuspended in PBS containing 1% formalin. Cells were acquired on a BD Biosciences LSRFortessa with 4 lasers (405, 488, 532, and 633 nm) and analyzed using FlowJo software (Tree Star Inc.). At least 55,000 events were acquired for each sample as described previously (57, 58).

### CD16 cross-linking NK cell activation assay.

Briefly, 1 million mononuclear cells isolated from LNs were mixed with anti-CD16 antibody (3G8) and goat anti-mouse IgG F(ab)2 for 6 hours at 37°C with 5 μg/mL brefeldin A (BD Biosciences) and 6 μg/mL of monensin (BD Biosciences). Control wells were incubated in the absence of the anti-CD16 antibody. After incubation, cells were surface stained with the following antibodies: anti-NKG2A/C, anti-CD4, anti-CD3, anti–HLA-DR, anti-CD20, anti-CXCR5, and anti-CD107a. Next, the cells were treated with Cytofix/Cytoperm permeabilization solution (BD Pharmingen) and stained for anti–IFN-γ and anti–TNF-α. Flow cytometry data were collected and analyzed with FlowJo.

### Degranulation assay with MHC class 1–devoid cells.

To evaluate the cytotoxic potential of LN NK cells, we used K562 target cells (obtained in-house), which lack MHC class I. Target cells were washed twice and plated in R-10 medium at a final concentration of 200,000 per well in U-bottom 5 mL stimulation tubes. After incubation, effector cells were washed and then added at the 20:1 effector-to-target (E/T) ratios to a final volume of 200 μL. Anti-CD107a antibody was added to the tubes prior to adding the effector (fresh effector LN cells [2 × 10^6^]) and target cells. Tubes were incubated at 37°C for 6 hours. One hour after initial stimulation, Golgi Stop and Golgi Plug were added to capture the intracellular cytokines. After incubation, cells were stained with LIVE/DEAD viability dye (Invitrogen) and appropriate surface and intercellular staining. Tubes were washed twice with FACS wash and finally resuspended in 200 μL of a 2% PBS–paraformaldehyde solution. Tubes were stored at 4°C until acquisition on a LSRII machine equipped with a high-throughput system (BD Biosciences). Cytotoxicity was measured by frequency of CD107a, IFN-γ, and TNF-α production by effector NK cells. LN cells cultured in the absence of target cells were used as negative controls to correct for background levels. To evaluate the cytotoxic potential of LN NK cells, we used 721.221 target cells (obtained in-house), which lack MHC class I. Target cells were washed twice and plated in R-10 medium at a final concentration of 200,000 per well in U-bottom 5 mL stimulation tubes. After incubation, effector cells were washed and then added at the 10:1 E/T ratios to a final volume of 200 μL. Anti-CD107a antibody was added to the tubes prior to adding the effector (fresh effector LN cells [1 × 10^6^]) and target cells. Tubes were incubated at 37°C for 6 hours. One hour after initial stimulation, Golgi Stop and Golgi Plug were added to capture the intracellular cytokines. After incubation, cells were stained with LIVE/DEAD viability dye (Invitrogen) and appropriate surface and intercellular staining. Tubes were washed twice with FACS wash and resuspended in 200 μL of a 2% PBS–paraformaldehyde solution and acquired in LSRII. Cytotoxicity was measured by frequency of CD107a production by effector NK cells. LN cells cultured in the absence of target cells were used as negative controls to correct for background levels.

### FACS cell sorting.

Mononuclear cells isolated from LNs were stained with anti-CD3, anti-CD8, anti-NKG2A, anti–HLA-DR, anti-CD20, and anti-CD95. Sorting of CXCR5^+^ and CXCR5^–^ NK cells was performed using a FACS Aria (BD Biosciences). Post-sorting FACS analysis determined that sorted NK cell subsets were on average more than 98% pure.

### Confocal microscopy.

Tissue imaging of OCT compound–embedded tissue sections was performed as described previously (35). Briefly, fresh LN biopsies from SHIV-infected rhesus macaques were fixed in 4% PFA for 6 hours at room temperature followed by embedding in OCT compound and freezing. Next, 8–10 μm thick sections were sectioned and quickly fixed in 70% acetone and 30% PFA mix. Fixed sections were used for immunostaining as described here. Sections were blocked with PBS carrying 2% BSA, 2% donkey serum, and 0.025% Triton X-100 for 1 hour at room temperature. After 3 washes with chilled PBS, tissue sections were subjected to overnight incubation with primary antibodies in 1% BSA and 0.025% Triton X-100–supplemented PBS. The primary antibodies contained rat anti-human CD3 (Bio-Rad, MCA1477), rabbit anti-NKG2A (Abcam, ab93169), goat anti-hCD20 (LSBio, LS-B11144), and mouse anti-CXCR5 (NIH), or mouse α granzyme B (clone GB11, Invitrogen, MA1-80734). The next day, primary antibodies were washed off using chilled PBS followed by incubation with the secondary antibodies for 1 hour at room temperature. The secondary antibody cocktail contained anti-rat IgG–Alexa Fluor 647, anti-mouse IgG–Alexa Fluor 555, anti-goat IgG–Alexa Fluor 405, and anti-rabbit–Alexa Fluor 488. Slides were washed thrice with chilled PBS and mounted using antifade mounting media followed by confocal imaging. Imaging was performed on Olympus FV1000 using 20× objective, and images were analyzed using ImageJ (NIH).

### RNA-Seq and downstream analyses.

RNA was extracted from sorted CXCR5^+^ and CXCR5^–^ cells from LNs stored at −80°C in RLT buffer with 1% 2-ME using RNeasy Mini kits (QIAGEN) with DNase digest and QIAcube automation stations. Extracted RNA was quantified using a NanoDrop 2000 spectrophotometer (Thermo Fisher Scientific) and the quality was assessed by Bioanalyzer analysis (Agilent Technologies). Ten nanograms of total RNA was used as input for cDNA amplification using 5′ template-switch PCR with the Clontech SMART-Seq v4 Ultra Low Input RNA kit. Amplified cDNA was fragmented and appended with dual-indexed barcodes using Illumina Nextera XT DNA Library Prep kits. The amplified libraries from both sets were validated by capillary electrophoresis on the Agilent 4200 TapeStation. The libraries were normalized, pooled, and sequenced on the Illumina HiSeq 3000 system employing a single-end 101-cycle run at average read depths of 30 × 10^6^ reads per sample. Reads were mapped to the MacaM version 7 assembly of the Indian rhesus macaque genomic reference (59) with RhesusGenome using STAR (version 2.5.2b) with default alignment parameters (60) (https://www.unmc.edu/rhesusgenechip/index.htm). Abundance estimation of raw read counts per transcript was done internally with STAR using the HTSeq-count algorithm (61). As defined previously (PMID 34237254), these transcript counts were normalized by TMM (trimmed mean of M values) by correcting for library sizes. Where required, differential expression per gene (versus the outcome of interest; i.e., CXCR5 MFI or CXCR5+/– groups) was computed using generalized linear modeling (edgeR package in R). To assess enrichment of pathways, gene sets were obtained from the c2 module of the Molecular Signatures Database (MSigDB) (v7.4). Next, fGSEA (from the fGSEA package in R) on a pre-ranked table of genes (sorted by log-fold change) was used to compute enrichment of each pathway and define leading-edge genes that drove the enrichment. Finally, sample-level enrichment analyses were then done to concatenate leading-edge genes into a per-sample score, and these scores were presented as heatmaps. Unless specified otherwise, all transcriptome analyses were conducted in R, and *P* values less than 0.05 were considered significant.

### Cytokine assays.

Meso Scale Discovery Multi-Array Technology was used for cytokine evaluation. A cytokine panel containing the following analytes was screened: IL-15, IL-7 as per manufacturer’s instructions, using 25 μL of each specimen from each macaque in duplicates. The results were extrapolated from the standard curve from each specific analyte and plotted in pg/mL for cytokines, respectively, using DISCOVERY WORKBENCH v4.0 software (Meso Scale Discovery). IL-12p70 ELISA was performed using Rhesus Macaque IL-12p70 ELISA kit from Raybiotech (ELK-IL12p70-1).

### Quantitation of SHIV RNA.

The SHIV copy number in plasma was determined using quantitative real-time PCR as described previously (57).

### Statistics.

A paired 2-tailed *t* test was used for comparisons between 2 or more subsets from the same animal. An unpaired *t* test was used for comparisons between SHIV-uninfected and SHIV-infected animals. Spearman’s rank test was used for all correlations. Boolean analyses were performed using SPICE software (National Institute of Allergy and Infectious Diseases, NIH). GraphPad Prism was used to determine *P* values. *P* values of less than 0.05 were considered significant.

### Study approval.

All animal procedures were approved by the IACUC and Institutional Biosafety Committee of Emory University, Atlanta, Georgia, USA, and were performed in strict accordance with NIH guidelines. The YNPRC facilities are accredited by the American Association for Accreditation of Laboratory Animal Care and licensed by the US Department of Agriculture.

## Author contributions

VV contributed to the concept and design of experiments. VV and RRA supervised the macaque projects and coordinated the experiments. SAR, TMS, SG, US, HB, SPR, SAA, CI, and VV performed experiments, and SAR, VV, US, and CI analyzed the data. JMB, AAS, GKT, RPJ, RPS, and SEB provided help with RNA-Seq and data analysis. SG, CI, US, and SNW provided technical help with in vitro experiments with cytokines. FV and SAA provided IL-12 and IL-15 cytokines. SAR and VV wrote the manuscript, and SAR, VV, and SNW edited the manuscript. All authors reviewed the manuscript and discussed the work.

## Supplementary Material

Supplemental data

## Version 1 03/10/2022

In-Press Preview

## Version 2 04/22/2022

Electronic publication
