# Supplementary material for: Lymph node CXCR5+ NK cells associate with control of chronic SHIV infection
Source: JCI Insight. 2022 Apr 22;7(8):e155601. doi: 10.1172/jci.insight.155601 (PMC9089783; doi:10.1172/jci.insight.155601)
Supplement: Supplemental data [file jciinsight-7-155601-s046.pdf]

Supplementary Figures and Legends:

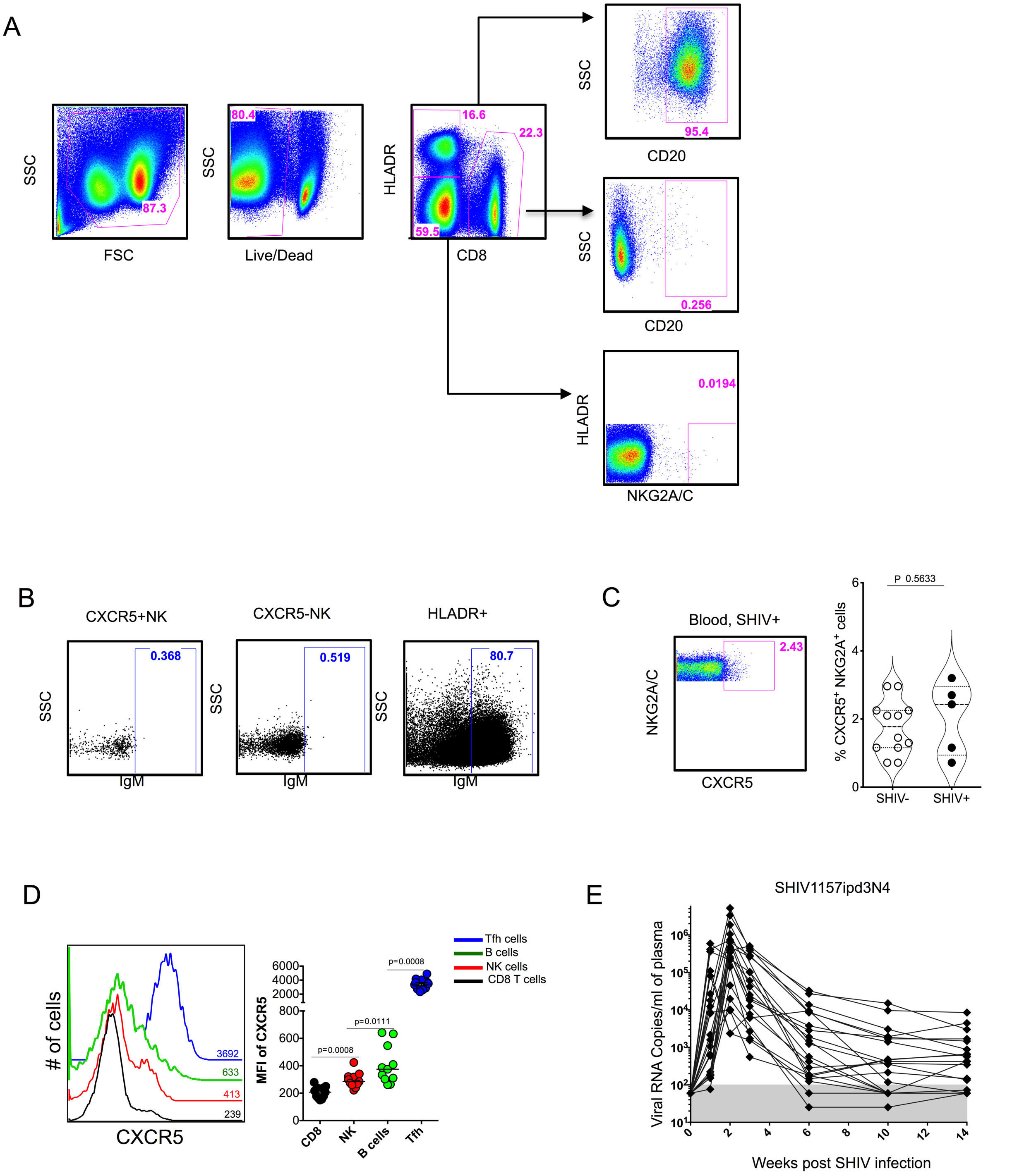

Supplemental Figure 1. (A) Gating Strategy for identifying CXCR5+ NK cells in the lymph node. (B) Representative flow plot display there is no IgM staining in NK cell subsets. Only HLADR+ cells are IgM+ cells in the macaque LN. (C) Representative flow plot display the expression of CXCR5 in blood NK cells. (D) MFI of CXCR5 on different subsets of lymph node cells. NK cells express higher levels of CXCR5 than CD8 T cells. (E) Viral RNA levels of macaques infected with SHIV1157ipd3N4 (n=21). Mann-Whitney test was used for comparison analysis.

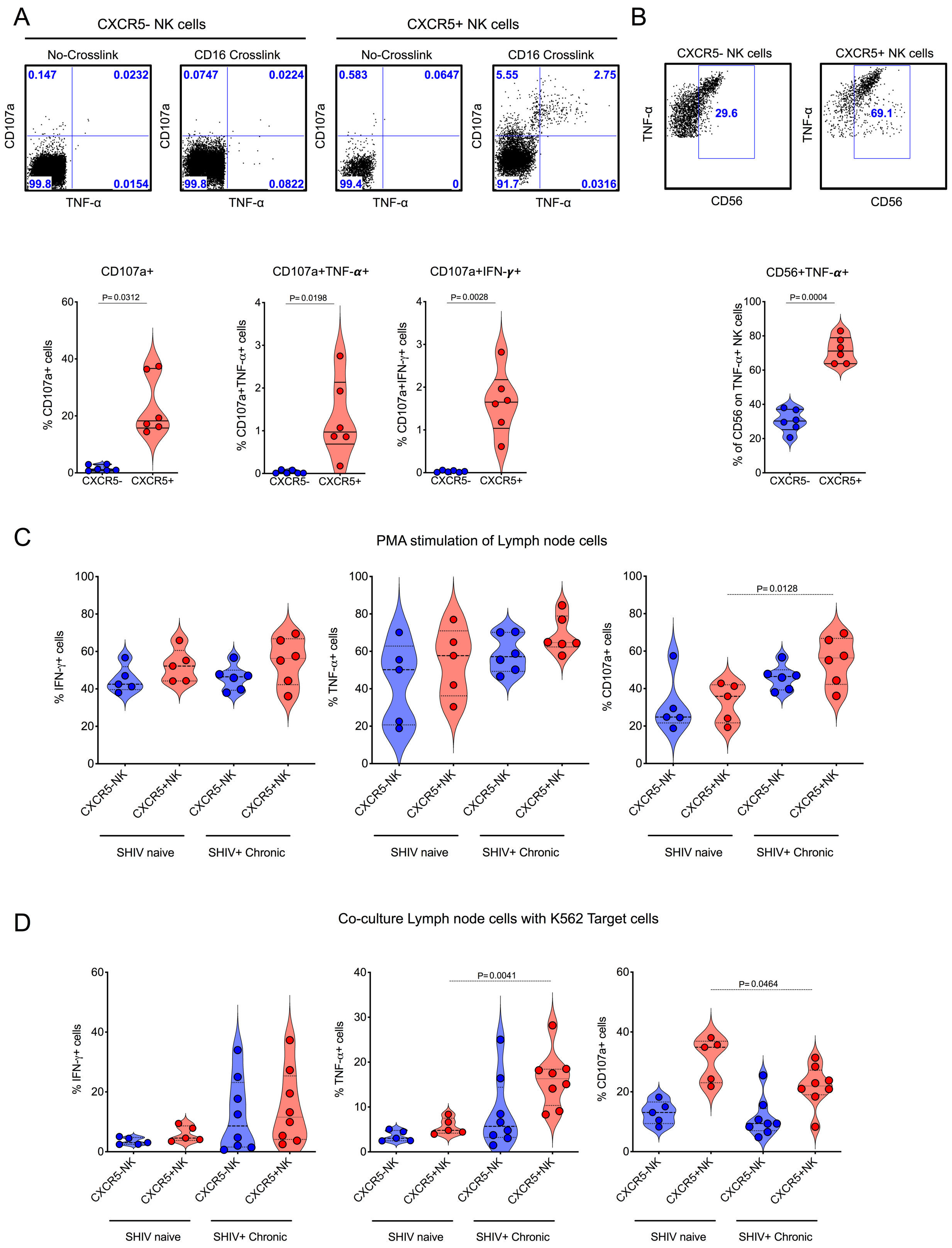
A
CXCR5- NK cells
CXCR5+ NK cells
No-Crosslink
CD16 Crosslink
B
CXCR5- NK cells
CXCR5+ NK cells
CD107a+
CD107a+TNF-α+
CD107a+IFN-γ+
CD56+TNF-α+
P=0.0312
P=0.0198
P=0.0028
P=0.0004
CXCR5-
CXCR5+
C
PMA stimulation of Lymph node cells
P=0.0128
CXCR5-NK
CXCR5+NK
SHIV naive
SHIV+ Chronic
D
Co-culture Lymph node cells with K562 Target cells
P=0.0041
P=0.0464

7

Supplemental Figure 2. (A) CD16 crosslinking experiment on LN cells. CXCR5+NK cells express higher levels of cytokines (n=6). (B) CD56 expression is higher on the cytokine+ CXCR5+ NK cells. Gating Strategy for identifying CXCR5$^+$ NK cells in the lymph node. (C) Cytokine expression and degranulation (CD107a$^+$ staining) profiles of CXCR5$^+$ and CXCR5$^-$ NK cells following 6-hours ex vivo culture in presence (PMA) or absence (NS) of PMA and ionomycin determined by intracellular cytokine staining and flow cytometry with SHIV naïve (n=5) and SHIV chronically infected animals (n=6). (D) Similar assessment of cytokine expression and degranulation of CXCR5$^+$ and CXCR5$^-$ NK cells following 6-hours co-culture with MHC class I devoid K562 cells comparing SHIV naïve (n=5) and SHIV infected animals (n=8). Wilcoxon's matched-pairs signed rank test was used to compare the frequencies of CXCR5$^+$ NK cells between blood and lymph node.

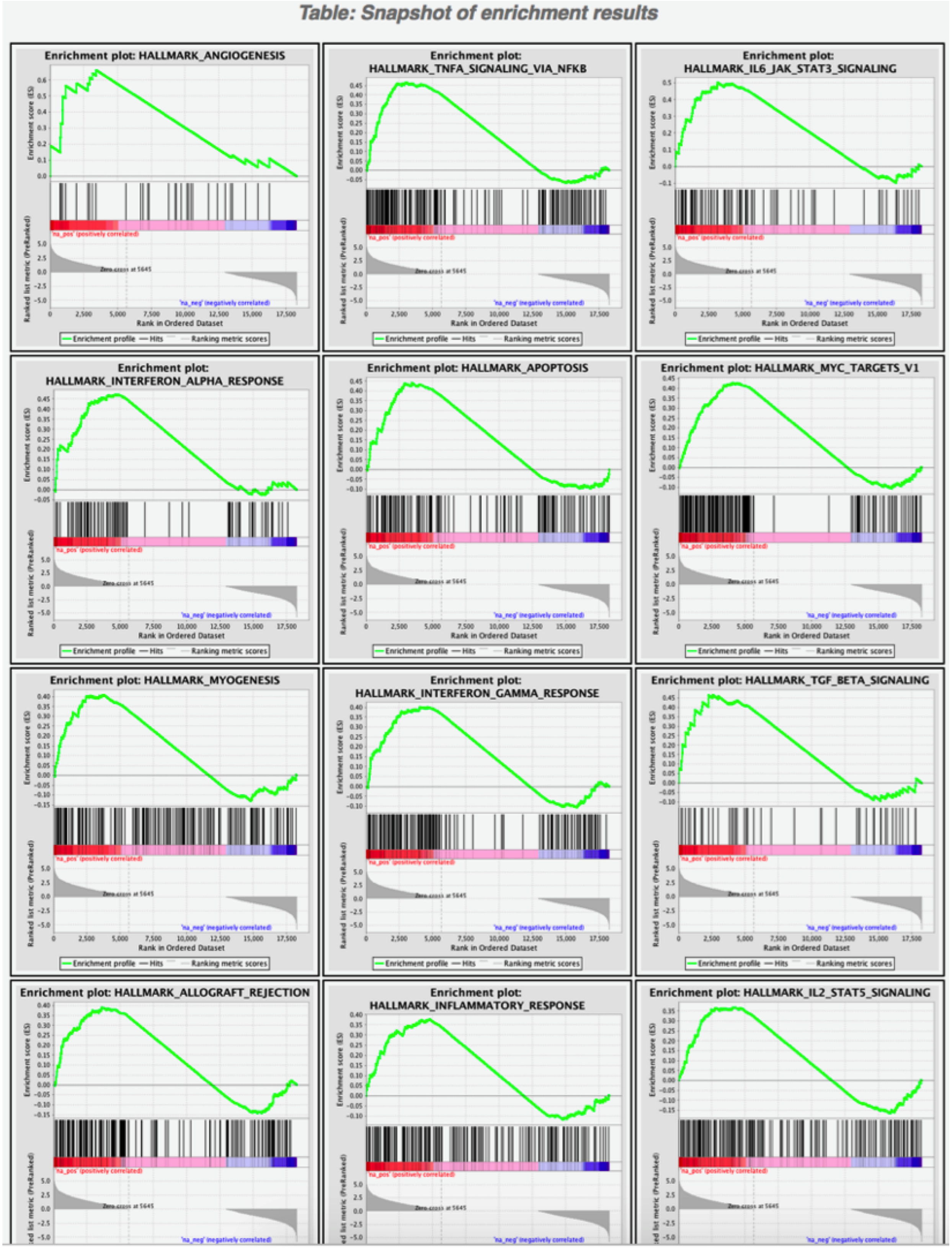
Table: Snapshot of enrichment results
Enrichment plot: HALLMARK_ANGIOGENESIS
Enrichment plot: HALLMARK_TNFA_SIGNALING_VIA_NFKB
Enrichment plot: HALLMARK_IL6_JAK_STAT3_SIGNALING
Enrichment plot: HALLMARK_INTERFERON_ALPHA_RESPONSE
Enrichment plot: HALLMARK_APOPTOSIS
Enrichment plot: HALLMARK_MYC_TARGETS_V1
Enrichment plot: HALLMARK_MYOGENESIS
Enrichment plot: HALLMARK_INTERFERON_GAMMA_RESPONSE
Enrichment plot: HALLMARK_TGF_BETA_SIGNALING
Enrichment plot: HALLMARK_ALLOGRAFT_REJECTION
Enrichment plot: HALLMARK_INFLAMMATORY_RESPONSE
Enrichment plot: HALLMARK_IL2_STAT5_SIGNALING
Enrichment score (ES)
Ranked list metric (PreRanked)
Rank in Ordered Dataset
Enrichment profile
Hits
Ranking metric scores

Supplemental Figure 3. Snap shot of GSEA plots enriched in CXCR5+ NK cells compared with CXCR5- NK cells in the LN of chronically SHIV infected animals (n=4).

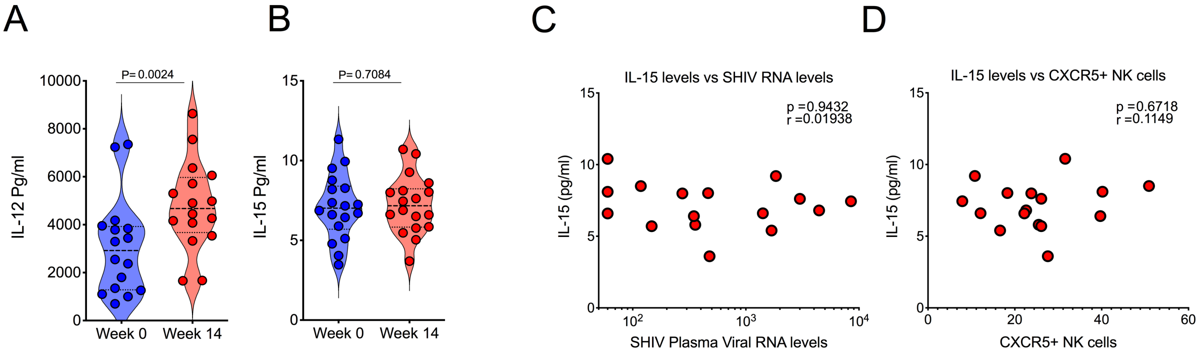

Supplemental Figure 4. (A) Plasma level concentration of IL-12p70 in pg/ml comparing SHIV naïve and SHIV infected animals (n=15), data display paired samples from uninfected and infected time points. (B) Plasma level concentration of IL-15 in pg/ml comparing SHIV naïve and SHIV infected animals, data display paired samples from uninfected and infected time points. (C) Correlation between plasma levels of IL-15 cytokine with SHIV RNA levels (n=15). (D) Correlation between plasma levels of IL-15 cytokine with CXCR5+ NK cells (n=15). Mann-Whitney test was used invitro analysis. Pearson correlation was used for correlation analysis.

Supplemental Table: 1. This table display cohort of SHIV infected macaques used in this study. A total of 22 animals have been studied.

Supplemental Table: 2. This table display antibodies and reagents used in this study.

Supplemental Table: 3. This table display gene set enrichment analyses to probe for alternations in major cytokine/chemokine genesets (obtained from Reactome database)

Supplemental Table: 4. This table display genesets were significantly associated (p-value <0.05; with the CXCR5 MFI across sorted CXCR5$^+$ and CXCR5- NK cell samples.

Supplemental Table 1. Cohort of SHIV infected rhesus macaques used for the study

Animal Name	Set-Point Viral Load (#)	Challenge Virus	Route of Infection	Mamu A01 Status	Sex
RFd15	60	SHIV1175	Intra-rectal	-	Female
RCf15	478	SHIV1175	Intra-rectal	-	Male
RSp15	464	SHIV1175	Intra-rectal	+	Female
RWk14	275	SHIV1175	Intra-rectal	-	Female
RGp15	429	SHIV1175	Intra-rectal	-	Male
RYc15	4440	SHIV1175	Intra-rectal	-	Male
RHv14	118	SHIV1175	Intra-rectal	-	Male
RBr15	6	SHIV1175	Intra-rectal	-	Male
RCe15	1843	SHIV1175	Intra-rectal	+	Male
RRi15	35	SHIV1175	Intra-rectal	+	Male
RJc15	418	SHIV1175	Intra-rectal	-	Female
RKi15	60	SHIV1175	Intra-rectal	+	Male
RSo15	1413	SHIV1175	Intra-rectal	-	Male
218-12	347	SHIV1175	Intra-rectal	-	Male
RUc16	1688	SHIV1175	Intra-rectal	-	Male
RJv15	60	SHIV1175	Intra-rectal	-	Female
RFj15	60	SHIV1175	Intra-rectal	-	Female
127_12	60	SHIV1175	Intra-rectal	-	Female
RHr15	147	SHIV1175	Intra-rectal	+	Male
RWn15	60	SHIV1175	Intra-rectal	+	Male
RJy15	8490	SHIV1175	Intra-rectal	-	Male
RKe15	3005	SHIV1175	Intra-rectal	+	Male
REr18	-	-	-	+	Male
RRq18	-	-	-	+	Male
RUw19	-	-	-	+	Male
RTj18	-	-	-	+	Male
RSi18	-	-	-	-	Male

Supplemental Table: 2 List of antibodies used for staining blood and lymph nodes

MARKER	COLOR	CLONE	COMPANY
CD3	BUV395	SP34-2	BD Biosciences
CCR7	BV785	150503	R & D System
CD8α	PerCp	T8/7Pt-3F9	NHP Resource Program
CD8α	PerCp	SK1	Bio Legend
CD16	BV650	3G8	BD Biosciences
CD56	PEcy7	NCAM16.2	BD Biosciences
NKG2A	PE	Z199	Beckman-Coulter
CD20	Alexa-700	2H7	BD Biosciences
HLA-DR	Per-Cp	G46-6	BD Biosciences
CXCR3	BV605	IC6	BD Biosciences
CCR6	Amcyan	11A9	BD Biosciences
CCR7	FITC	150503	R & D System
CCR4	Petexred	1G1	BD Biosciences
CD32a	PE	FL18.26	BD Biosciences
CD69	Alex-700	FN50	BD Biosciences
Ki67	PeCy7	B56	BD Biosciences
Perforin	FITC	Mab-Pf344	MabTech
Granzyme-B	Alex-700	GB-11	BD Biosciences
BCL-6	FITC	K112-91	BD Biosciences
IFN-g	Alexa-700	B27	BD Biosciences
TNF-a	PEtex-red	Mab11	BD Biosciences
CD107a	FITC	H4A3	BD Biosciences
L/D	APC H7	Fixable Near IR stain	Invitrogen
CXCR5	PE		NHP Resource Program

FASL	PECy7	NOK-1	Biolegend
Anti-CD16	Purified	3G8	Biolegend
**OTHER REAGENTS**			
**REAGENT**	**CATALOGUE**		**COMPANY**
Cytofix/Cytoperm	51-2090KZ		BD Biosciences
BD Perm/Wash	51-2091KZ		BD Biosciences
BD FACS™ Lysing solution	349202		BD Biosciences
BD FACS™ Permeabilizing solution 2	340973		BD Biosciences
Fixtion/Permeabilization concentrate	00-5123-43		Invitrogen
Fixation Perm Diluent	00-5223-56		Invitrogen
Permeabilization Buffer	00-8333-56		Invitrogen

Supplemental Table: 3: Gene set enrichment analysis obtained from reactome database

Geneset	Source
REACTOME_CHEMOKINE_RECEPTORS_BIND_CHEMOKINES	http://www.gsea-msigdb.org/gsea/msigdb/cards/REACTOME_CHEMOKINE_RECEPTORS_BIND_CHEMOKINES
REACTOME_SIGNALING_BY_GPCR	http://www.gsea-msigdb.org/gsea/msigdb/cards/REACTOME_SIGNALING_BY_GPCR
REACTOME_CLASS_I_MHC_MEDIATED_ANTIGEN_PROCESSING_PRESENTATION	http://www.gsea-msigdb.org/gsea/msigdb/cards/REACTOME_CLASS_I_MHC_MEDIATED_ANTIGEN_PROCESSING_PRESENTATION
REACTOME_ANTIGEN_PROCESSING_CROSS_PRESENTATION	http://www.gsea-msigdb.org/gsea/msigdb/cards/REACTOME_ANTIGEN_PROCESSING_CROSS_PRESENTATION
REACTOME_MHC_CLASS_II_ANTIGEN_PRESENTATION	http://www.gsea-msigdb.org/gsea/msigdb/cards/REACTOME_MHC_CLASS_II_ANTIGEN_PRESENTATION
REACTOME_INTERLEUKIN_6_SIGNALING	http://www.gsea-msigdb.org/gsea/msigdb/cards/REACTOME_INTERLEUKIN_6_SIGNALING
REACTOME_INTERLEUKIN_7_SIGNALING	http://www.gsea-msigdb.org/gsea/msigdb/cards/REACTOME_INTERLEUKIN_7_SIGNALING
REACTOME_INTERLEUKIN_17_SIGNALING	http://www.gsea-msigdb.org/gsea/msigdb/cards/REACTOME_INTERLEUKIN_17_SIGNALING
REACTOME_INTERLEUKIN_3_INTERLEUKIN_5_AND_GM_CSF_SIGNALING	http://www.gsea-msigdb.org/gsea/msigdb/cards/REACTOME_INTERLEUKIN_3_INTERLEUKIN_5_AND_GM_CSF_SIGNALING
REACTOME_INTERLEUKIN_10_SIGNALING	http://www.gsea-msigdb.org/gsea/msigdb/cards/REACTOME_INTERLEUKIN_10_SIGNALING
REACTOME_INTERLEUKIN_4_AND_INTERLEUKIN_13_SIGNALING	http://www.gsea-msigdb.org/gsea/msigdb/cards/REACTOME_INTERLEUKIN_4_AND_INTERLEUKIN_13_SIGNALING
REACTOME_INTERLEUKIN_15_SIGNALING	http://www.gsea-msigdb.org/gsea/msigdb/cards/REACTOME_INTERLEUKIN_15_SIGNALING
REACTOME_INTERLEUKIN_35_SIGNALLING	http://www.gsea-msigdb.org/gsea/msigdb/cards/REACTOME_INTERLEUKIN_35_SIGNALLING
REACTOME_INTERLEUKIN_9_SIGNALING	http://www.gsea-msigdb.org/gsea/msigdb/cards/REACTOME_INTERLEUKIN_9_SIGNALING
REACTOME_INTERLEUKIN_37_SIGNALING	http://www.gsea-msigdb.org/gsea/msigdb/cards/REACTOME_INTERLEUKIN_37_SIGNALING
REACTOME_INTERLEUKIN_18_SIGNALING	http://www.gsea-msigdb.org/gsea/msigdb/cards/REACTOME_INTERLEUKIN_18_SIGNALING
REACTOME_INTERLEUKIN_2_SIGNALING	http://www.gsea-msigdb.org/gsea/msigdb/cards/REACTOME_INTERLEUKIN_2_SIGNALING
REACTOME_INTERLEUKIN_12_SIGNALING	http://www.gsea-msigdb.org/gsea/msigdb/cards/REACTOME_INTERLEUKIN_12_SIGNALING
REACTOME_INTERLEUKIN_1_SIGNALING	http://www.gsea-msigdb.org/gsea/msigdb/cards/REACTOME_INTERLEUKIN_1_SIGNALING
REACTOME_INTERLEUKIN_23_SIGNALING	http://www.gsea-msigdb.org/gsea/msigdb/cards/REACTOME_INTERLEUKIN_23_SIGNALING
REACTOME_INTERLEUKIN_27_SIGNALING	http://www.gsea-msigdb.org/gsea/msigdb/cards/REACTOME_INTERLEUKIN_27_SIGNALING
REACTOME_INTERLEUKIN_21_SIGNALING	http://www.gsea-msigdb.org/gsea/msigdb/cards/REACTOME_INTERLEUKIN_21_SIGNALING
REACTOME_SIGNALING_BY_TGF_BETA_FAMILY_MEMBERS	http://www.gsea-msigdb.org/gsea/msigdb/cards/REACTOME_SIGNALING_BY_TGF_BETA_FAMILY_MEMBERS
REACTOME_TNF_SIGNALING	http://www.gsea-msigdb.org/gsea/msigdb/cards/REACTOME_TNF_SIGNALING

Supplemental Table: 4: Gene set enrichment analysis

pathway	pval	NES
REACTOME_CHEMOKINE_RECEPTORS_BIND_CHEMOKINES	0.00437351	1.714192456
REACTOME_INTERLEUKIN_12_SIGNALING	0.01250788	1.614360914
REACTOME_INTERLEUKIN_35_SIGNALLING	0.02851897	1.526767284
REACTOME_INTERLEUKIN_7_SIGNALING	0.02926404	1.519492681
REACTOME_INTERLEUKIN_15_SIGNALING	0.05291971	1.498999658
REACTOME_ANTIGEN_PROCESSING_CROSS_PRESENTATION	0.00619051	-1.902149855

pathway	leadingEdge
REACTOME_CHEMOKINE_RECEPTORS_BIND_CHEMOKINES	CXCR3 CCL5 CCR6 CCR7 CXCR5 XCL2
REACTOME_INTERLEUKIN_12_SIGNALING	MSN JAK1 CAPZA1 HNRNPF ARF1 RAP1B LCP1 AIP CNN2 IL12RB1 IL12RB2 JAK2
REACTOME_INTERLEUKIN_35_SIGNALLING	JAK1 STAT3 IL27RA IL12RB2 JAK2 EBI3 STAT4
REACTOME_INTERLEUKIN_7_SIGNALING	JAK1 IL2RG STAT3 STAT5A SOCS1 CISH JAK3
REACTOME_INTERLEUKIN_15_SIGNALING	JAK1 IL2RG STAT3 STAT5A IL15RA SOS2 JAK3 IL2RB SOS1
REACTOME_ANTIGEN_PROCESSING_CROSS_PRESENTATION	B2M UBC TAP1 PSMD1 UBB PSMA7 PSMB1 PSMB6 PSMD14 PSMD8 CTSS PSME1 MYD88 PSMA1 UBA52 PSMB7 CALR IKBKG VAMP8 RPS27A TIRAP
